# The Glass Transition: A Topological Perspective

**DOI:** 10.3390/e27030258

**Published:** 2025-02-28

**Authors:** Arthur Vesperini, Roberto Franzosi, Marco Pettini

**Affiliations:** 1 Department of Physical Sciences, Earth and Environment (DSFTA), University of Siena, Via Roma 56, 53100 Siena, Italy; arthur.vesperini@unisi.it (A.V.); roberto.franzosi@unisi.it (R.F.); 2 INFN Sezione di Perugia, 06123 Perugia, Italy; 3 Aix-Marseille University, Université de Toulon, CNRS, 13288 Marseille, France; 4 Centre de Physique Théorique, 13288 Marseille, France; 5 Quantum Biology Lab, Howard University, Washington, DC 20059, USA

**Keywords:** statistical mechanics, phase transitions, riemannian geometry, differential topology, glass former, Monte Carlo

## Abstract

Resorting to microcanonical ensemble Monte Carlo simulations, we study the geometric and topological properties of the state space of a model of a network glass-former. This model, a Lennard-Jones binary mixture, does not crystallize due to frustration. We have found two peaks in specific heat at equilibrium and at low energy, corresponding to important changes in local ordering. These singularities were accompanied by inflection points in geometrical markers of the potential energy level sets—namely, the mean curvature, the dispersion of the principal curvatures, and the variance of the scalar curvature. Pinkall’s and Overholt’s theorems closely relate these quantities to the topological properties of the accessible state-space manifold. Thus, our analysis provides strong indications that the glass transition is associated with major changes in the topology of the energy level sets. This important result suggests that this phase transition can be understood through the topological theory of phase transitions.

## 1. Introduction

As of today, the glass transition still stands as an open problem in contemporary physics. Indeed, as glasses are amorphous solids, no symmetry breaking is associated with this transition, which is therefore outside the domain of applicability of Landau theory [1]. It has further been argued that glass-forming materials (at least *strong* glass formers in Angell’s classification [2]) do not even exhibit a transition in the conventional sense; the transition temperature $T_{g}$ is usually regarded as purely conventional, and is defined by the crossing of a threshold value of the viscosity or relaxation time. The aim of the present work is to study the glass transition from a new perspective. Specifically, we consider the topological and geometric properties of the state space of a known glass-forming model.

In a number of references [3,4,5], it has been shown that glass transitions most likely correspond to a *geometric transition*. Specifically, the *critical* points of the potential energy were studied, particularly their instability index (i.e., the number of negative eigenvalues of the Hessian matrix). It was found that the average index density vanishes at the glass transition [6,7].

Yet it is known, from Morse theory [1,8], that any change in the stability indices of a function defined on a differentiable manifold, such as the potential energy, is accompanied by a change in the topology of this manifold.

Furthermore, the relatively recent topological theory of phase transitions [1,9,10,11] has unraveled a deep link between classical phase transitions and changes in the topology of the potential level sets (PLS) $\Sigma_{\Phi }$, i.e., the iso-energy hypersurfaces. One advantage of the topological theory of phase transitions is that it applies to small systems (mesoscopic and nanoscopic scales), thus escaping the *thermodynamic limit dogma* upon which the Yang–Lee theory of phase transitions is built. Furthermore, it applies to phase transitions that occur in the absence of symmetry-breaking (hence in the absence of a well-defined order parameter). This second point is of great interest to us, as the glass transition notoriously falls into the latter category. It is worth mentioning that several other approaches to phase transitions in small-*N* systems have been proposed in the past [12,13], motivated by various physical problems that called for such a theoretical basis.

These observations compel us to investigate glass-forming systems, resorting to a few elegant theorems linking topological invariants to geometric quantities such as the mean curvature.

The topological theory of phase transitions is usually defined at equilibrium, as it requires the study of the whole PLS, regardless of metastability and dynamical slowing down. This could be considered an important drawback in its application to the glass transition, which is considered an inherently out-of-equilibrium phenomenon. However, glass formation can be regarded as a transitional, metastable state, induced by the slowing down of the dynamics due to frustration during crystallization [14,15]. This phenomenon is necessarily rooted in the equilibrium properties of matter. We thus deem this study of a glass-former’s equilibrium properties relevant in principle. Moreover, in systems that do not exhibit a true crystalline phase due to a high degree of frustration, the global free-energy minimum (or, in the microcanonical ensemble, the global entropy maximum at a given energy) may very well be a disordered, glassy state. It seems that, in this situation, the glass phase is not considered an equilibrium state solely because it breaks ergodicity, with the system being stuck in potential wells for extensive amounts of time.

While this proposition holds from the definition of thermodynamic equilibrium, we argue for its reevaluation under some specific conditions. In this highly frustrated system, the PLS at low energy is partitioned into a multitude of isolated wells, which we cannot escape under realistic conditions. However, ergodicity can be artificially restored by resorting to various numerical tricks that allow the system to jump out from one well to another. In such a situation, the glass phase can be studied as if it were an equilibrium state, in the sense that measurements can be performed via state-space importance sampling. Provided that there is no possible crystalline configuration that can “corrupt” this sampling, and that only glass states can be found at the energy of interest, we can then say that we have somewhat performed equilibrium measurements on the glass phase.

To this end, the study of binary mixtures, governed by soft sphere or Lennard-Jones potentials, represents an appropriate choice, as these systems often exhibit a high degree of frustration. In fact, numerous previous studies of the glass transition and low-temperature anomalies associated with the glass phase have been applied to this particular class of systems [6,16,17,18,19,20,21]. Of course, a variety of models exhibit a glass phase, including spin systems and even, in some specific conditions, discrete variable systems [22].

In Section 3.1, we present the model of the glass-former under investigation. Section 2.1 is devoted to the presentation of geometric quantities that are linked to topology by a handful of useful theorems; we show how these quantities can be measured in our simulations. In Section 2.2, we present our numerical results. We have found two peaks in specific heat, signaling a two-step phase transition. Using analytic tools developed specifically for the microcanonical ensemble, we identify them as a second-order and a weakly first-order critical point. Furthermore, we show that these transitions correspond to jumps in bond-orientational order parameters, as well as modifications in the spatial distribution profiles; these critical points are thus accompanied by modifications in the short-range structural properties of the system. Finally, we observe singular behaviors in various geometric quantities, coinciding with the observed transition, conclusively implying an underlying change in the topology of the potential energy level sets.

## 2. Material and Methods

### 2.1. Geometric Signatures of Topological Changes

Entropy stems as a fundamental building block of thermodynamics. In particular, in the microcanonical ensemble, most macroscopic observables can be retrieved from its derivatives. Furthermore, a microcanonical ensemble classification of phase transitions has recently been proposed [23], analogous to the notorious classification of Ehrenfest, that heuristically associates first- and second-order phase transitions to discontinuity of the second and third derivatives, respectively, of the entropy.

In standard Hamiltonian systems such as ours, where *H* is a quadratic function of the momenta, the kinetic part of the canonical partition is known to be trivial, as it reduces to a constant factor. In the microcanonical ensemble, the dissociation of the kinetic and configurational parts of the partition function is somewhat less evident, but can nevertheless be performed through Laplace transform techniques [24]; in particular, this separation allows for the practical expression of the microcanonial probability density (A2). It results in the relevant information being entirely contained in the configurational entropy

(1) $$S\left(\varphi \right)=\frac{1}{3N}\log\int d\Gamma \;\Theta \left(\varphi -\Phi (\Gamma )\right),$$

where Θ(x) is the Heaviside step function, which vanishes for x<0 and equates one for x≥0, φ is the potential, Φ(Γ) the potential as a function of the system’s configuration Γ, and 3N the number of degrees of freedom. Yet the latter expression can be rewritten in terms of the PLS volumes [1,25]

(2) $$S\left(\varphi \right)=\frac{1}{3N}\log\int_{0}^{\varphi }d\phi \;\int_{\Sigma_{\phi }}\frac{d\sigma }{\left|\nabla \Phi \right|},$$

where **∇** is the gradient operator, dσ is the elementary volume induced by the immersion in $R^{3N}$ of the PLS $\Sigma_{\phi }$, and the hypersurface of dimension 3N−1 is defined as

(3) $$\Sigma_{\phi }=\left\{\Gamma \in \Omega |\Phi \left(\Gamma \right)=\phi \right\},$$

where Ω is the full state space, and ϕ is the fixed value defining the PLS. Finally, it has been shown that Equation (2) can be expressed in terms of topological invariants, namely, [1]

(4) $$S\left(\varphi \right)=\frac{1}{3N}\log\left[Vol\left(S_{1}^{3N-1}\right)\sum_{i=0}^{3N}b_{i}\left(\Sigma_{\varphi }\right)+R_{1}\left(\varphi \right)\right]+\frac{1}{N}\log R_{2}\left(\varphi \right),$$

where $R_{1},R_{2}$ are smooth functions of the potential, $Vol(S_{1}^{3N-1})$ is the volume of the unit ball of dimension 3N−1, and $b_{i}\left(\Sigma_{\varphi }\right)$ is the *i*th Betti number of the manifold $\Sigma_{\varphi }$. The Betti numbers are a set of integer topological invariants, related, for instance, to the number of connected components and *n*-dimensional holes in a given manifold. The complete set of Betti numbers uniquely defines the topology of the latter (see e.g., [1]).

Equation (4) highlights the dependence of the configurational entropy on topological aspects of the PLS, encoded in the Betti numbers $b_{i}$. This observation was at the root of the *topological hypothesis*, stating that the deep mathematical origin of a phase transition was to be found in a topological change of the PLS. It is worth noting that while any phase transition is rooted in a topological change, not all topological changes entail a phase transition.

In the present work, we thus aim to establish a correspondence of the glass transition with topological changes in the PLS.

Probing the topology of the high-dimensional manifolds that constitute the PLS is by no means a simple task. To our best knowledge, there exists no way of fully characterizing it by means of measurable average observables—namely, the tools accessible to us. For lack of a complete reconstruction of the topology of the submanifolds of interest, it is nevertheless possible to probe *topological changes*, which are, in the end, our true object of study. There fortunately exist a few theorems of differential topology that draw sufficiently strong links between geometrical and topological quantities, allowing us to observe, when they are present, sharp topological *changes*.

We now introduce, very roughly, a few notions of differential extrinsic geometry that will be useful to the development of our topological probing. For a more extensive development of this framework, we refer the reader to [1,25,26].

In order to alleviate our notations, we now drop the dependence in Γ.

We first present Pinkall’s theorem [27], relating the average dispersion of principal curvatures with the weighted sum of the Betti numbers

(5) $$\frac{\int_{\Sigma_{\phi }}\sigma_{\kappa }^{2}\left(\Gamma \right)d\Gamma }{\int_{\Sigma_{\phi }}d\Gamma }={\left[Vol\left(S_{1}^{D}\right)\sum_{i=1}^{D}{\left(\frac{i}{D-1}\right)}^{D/2-i}b_{i}\left(\Sigma_{\phi }\right)\right]}^{2/D}-r\left(\Sigma_{\phi }\right),$$

where *D* is the dimension of the manifold, $\sigma_{\kappa }^{2}=\langle \kappa_{i}^{2}\rangle -{\langle \kappa_{i}\rangle }^{2}$ is the dispersion of the principal curvatures $\kappa_{i}$, and $r(\Sigma_{\phi })$ is a remainder, which stays small provided that $\sigma_{\kappa }^{2}$ does not exhibit too large variations on the submanifold $\Sigma_{\phi }$.

Another geometric quantity that connects to topological invariants is the length $\Delta_{sec}$ of the range of the sectional curvatures. Overholt’s theorem [28] indeed states that it provides an upper bound to the sum of the Betti numbers

(6) $$\Delta_{sec}\geq {\left[\frac{Vol\left(S^{D}\right)\sum_{i=0}^{D}b_{i}\left(\Sigma_{\phi }\right)}{2Vol(\Sigma_{\phi })}\right]}^{2/D}.$$

In turn, $\Delta_{sec}$ is related to the variance of the scalar curvature $R_{\Sigma }$, as the latter is simply defined as the sum of all the sectional curvatures at a given point Γ

(7) $$R_{\Sigma }=\sum_{i\neq j}K_{ij}=\sum_{i\neq j}\kappa_{i}\kappa_{j},$$

where $K_{ij}$ is the sectional curvatureof sectional plane $\left(u_{i},u_{j}\right)$, and ${\left\{u_{j}\right\}}_{j=1,\cdots ,D}$ forms an orthonormal basis in the tangent space at this point.

It results in

(8) $$\frac{\langle R_{\Sigma }^{2}\rangle -{\langle R_{\Sigma }\rangle }^{2}}{N(N-1)}\approx \Delta_{sec}\;,$$

To the best of our knowledge, the simplest way to compute these quantities in the context of numerical simulations is by considering the *Weingarten operator*, also called the *shape operator*. A most useful tool for characterizing the extrinsic geometry of hypersurfaces, it is the operator such that, for X∈TΣ a vector field in the tangent bundle of Σ, we have

(9) $$W_{n}\left(X\right)=-\nabla_{X}n,$$

where $\nabla_{X}$ is the Levi-Civita connexion on Σ, and

$$n=\frac{\nabla \phi }{\left|\nabla \phi \right|}\left(\Gamma \right)$$

is the normal to Σ at a given point Γ.

The trace of the shape operator and of its square can be expressed in terms of mere derivatives of Φ, namely

(10) $$\begin{array}{c} \begin{array}{cc} \mathrm{Tr}\left[W_{n}\right] & =\frac{\Delta \Phi }{\left|\nabla \Phi \right|}-\frac{\nabla \Phi^{*}\cdot \mathrm{Hess}\left(\Phi \right)\cdot \nabla \Phi }{{\left|\nabla \Phi \right|}^{3}}\\ \mathrm{Tr}\left[W_{n}^{2}\right] & =\frac{\mathrm{Tr}\left[\mathrm{Hess}{\left(\Phi \right)}^{2}\right]}{{\left|\nabla \Phi \right|}^{2}}+\frac{{\left|\nabla \Phi^{*}\cdot \mathrm{Hess}\left(\Phi \right)\cdot \nabla \Phi \right|}^{2}}{{\left|\nabla \Phi \right|}^{6}}-2\frac{{\left|\mathrm{Hess}\left(\Phi \right)\cdot \nabla \Phi \right|}^{2}}{{\left|\nabla \Phi \right|}^{4}}, \end{array} \end{array}$$

where ΔΦ and $\mathrm{Hess}\left(\Phi \right)$ denote the Laplacian and the Hessian of ϕ, and “·” the scalar product, respectively.

The eigenvalues of $W_{n}$ are the *D* principal curvatures $\kappa_{i}$. As a result, the above-mentioned geometric quantities can all be expressed with combinations of $\mathrm{Tr}\left[W_{n}\right]$ and $\mathrm{Tr}\left[W_{n}^{2}\right]$, namely,

(11) $$\begin{array}{cc} M_{\Sigma } & =\frac{\mathrm{Tr}\left[W_{n}\right]}{D}\\ \sigma_{\kappa }^{2} & =\frac{\mathrm{Tr}\left[W_{n}^{2}\right]}{D}-\frac{\mathrm{Tr}{\left[W_{n}\right]}^{2}}{D^{2}}\\ R_{\Sigma } & =\mathrm{Tr}{\left[W_{n}\right]}^{2}-\mathrm{Tr}\left[W_{n}^{2}\right], \end{array}$$

where $M_{\Sigma }$ is the total mean curvature. The combination of Formulas (10) and (11) clearly provides a straightforward way to obtain the quantities of interest in the context of numerical simulations by simply computing and combining the gradient and Hessian of the potential function Φ at each measurement step.

The latter geometric quantities pertain to the geometrical characteristics of the PLS, while our simulations are performed at constant total energy *E*. However, at large *N*, the fluctuations in Φ and *K* tend to vanish, and the surfaces of constant $K\left({\left\{p_{i}\right\}}_{i=1,\cdots ,N}\right)=\sum_{i}p_{i}^{2}/2$ are diffeomorphic to 3N-hyperspheres; the energy level sets can then be seen as product manifolds $\Sigma_{E}∼S_{K}^{3N}\times \Sigma_{\phi }$. We thus consider ϕ(E) stable enough for the corresponding PLSs to be diffeomorphic to one another, and for the general behavior of the above-defined geometric quantities to be trusted.

### 2.2. Numerical Methods

Using a microcanonical ensemble Monte Carlo scheme described in Appendix A, we explored the behavior of the model defined by (12).

We simulated a system of size N=216 particles. Periodic boundary conditions and smooth cutoffs have been implemented, as described in Appendix B.

It is worth noting that this simulations was, as is often the case in so-called glassy systems, very time-consuming and hard to equilibrate.

An exact estimation of the total computation time is difficult to assert in practice, partly due to the fact that the set of energies we considered was changed multiple times during this extensive work. To provide a rough idea of the involved time scales, the equilibration phase, added with the earlier simulations aimed at code-testing and optimization, took more than 400 h in CPU time per replica, with 50 replicas of the system. The *efficient computation time*, over which we performed retrieved the equilibrium data presented in this work, was 175 h per replica, with 100 replicas.

Throughout the following sections of this article, we present data acquired at equilibrium over $5.7\cdot 10^{6}$ *Monte Carlo sweeps* (that is, one Monte Carlo step per particle), with a sampling rate of 1/1000. Replica exchanges were attempted every 2000 sweeps.

## 3. Results

### 3.1. Model

The system we chose to consider here consists of a binary Lennard-Jones mixture, first introduced in [29], of Hamiltonian $H\left({\left\{q_{i}\right\}}_{i=1,\cdots ,N}\right)=K+\Phi \left({\left\{q_{i}\right\}}_{i=1,\cdots ,N}\right)$, where *K* is the total kinetic energy, the $q_{i}$ are the *N* three-dimensional position variables, and

(12) $$\begin{array}{cccccc} \Phi (\Gamma )= & \Phi_{11}\left(\Gamma \right) & + & \Phi_{22}\left(\Gamma \right) & + & \Phi_{12}\left(\Gamma \right)\\ = & \sum_{i,j\in \Lambda_{1}}4\epsilon_{11}{\left(\frac{\sigma_{11}}{r_{ij}}\right)}^{12}\; & + & \sum_{i,j\in \Lambda_{2}}4\epsilon_{22}{\left(\frac{\sigma_{22}}{r_{ij}}\right)}^{12}\; & + & \sum_{\begin{array}{c} i\in \Lambda_{1},\\ j\in \Lambda_{2} \end{array}}4\epsilon_{12}\left[{\left(\frac{\sigma_{12}}{r_{ij}}\right)}^{12}-{\left(\frac{\sigma_{12}}{r_{ij}}\right)}^{6}\right], \end{array}$$

where we introduced the shorthand notation $\Gamma ={\left\{q_{i}\right\}}_{i=1,\cdots ,N}$ for the instantaneous configuration of the system, $\Lambda_{1},\;\Lambda_{2}$ are the set of particles belonging to species 1 and 2, respectively, and $r_{ij}=\left|q_{i}-q_{j}\right|$. In this model, the interaction between particles of the same species is evidently purely short-range and repulsive, while that between particles of different species possesses an additional longer-ranged attractive component. These two types of interactions correspond to two soft sphere potentials and a Lennard-Jones potential, according to the classification proposed in [3]. The interaction parameters are set as

$$\begin{array}{cccc} \sigma_{22}/\sigma_{11} & =0.85\; & \sigma_{12}/\sigma_{11} & =0.49\\ \epsilon_{12}/\epsilon_{11} & =6\; & \epsilon_{22}/\epsilon_{11} & =1, \end{array}$$

and $\epsilon_{11}=1$, $\sigma_{11}=1$. The density ρ=1.6 and the respective concentrations of the two species are $c_{1}\approx 0.33$ and $c_{2}\approx 0.67$. Specifically, our numerical study considered a sample of $N_{1}=75$ particles of species 1 and $N_{2}=145$ particles of species 2, for a total of N=216 particles. As is customary, we set the Boltzmann constant as $k_{B}=1$. This set of parameters was proposed in [18] as appropriately reproducing the network glass-forming properties of amorphous silica.

In this numerical study, we do not take into account the microscopic details of the kinetic energy *K*; in the Monte Carlo scheme we employed, described in Appendix A, *K* is in fact employed as a “*demon*”, allowing us to keep the total energy *E* constant.

### 3.2. Characterization of the Phase Transition

As a preliminary to our analysis of the geometry and topology of the PLS, we show here the that a phase transition is indeed occurring, and try to determine its precise nature.

To this end, we examined quantities that are usually expected to exhibit singular behavior at the transition.

#### 3.2.1. Specific Heat, Caloric Curve, and Entropy Derivatives

The specific heat $c_{v}$ typically displays these critical behaviors in most phase transitions; this can be due to the presence of latent heat, in the case of a first-order phase transition, or to critical fluctuations, in the case of continuous phase transitions.

In the microcanonical ensemble, the specific heat can be computed according to

(13) $$c_{v}={\left(\frac{dT}{dE}\right)}^{-1},$$

where $T=\frac{2\langle K\rangle }{3N}$ is the kinetic temperature. Alternatively, we also use the results of [24], which used the Laplace-transform techniques to propose a variety of alternative definitions for usual thermodynamical observables. Among three different formulas for $c_{v}$, we only display one here, as they all yield the same results, up to the accessible precision.

(14) $$c_{v}=\frac{3}{2}{\left[1-\frac{3N}{2}\left(\frac{\langle K^{2}\rangle }{{\langle K\rangle }^{2}}-1\right)\right]}^{-1}.$$

The comparison of the curves obtained with both Equations (13) and (14) is commonly employed as an equilibration test (see, for instance, [30]).

**Figure 1:**
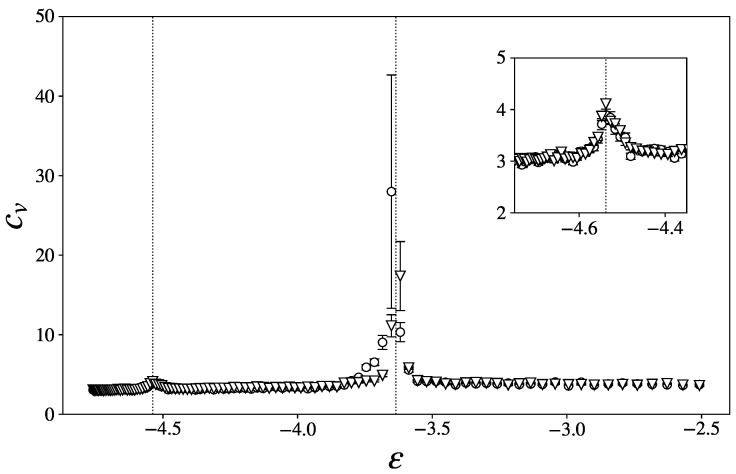
Specific heat $c_{v}$ as a function of the energy density ϵ, in a system with N=216. The blue circles were obtained using Equation (14), while the red triangles were obtained using Equation (13). The inset is a zoom on the low-energy peak. The dashed line is an arbitrary fit, meant to guide the eye.

Inspection of Figure 1 shows two clear peaks of the specific heat. These “anomalies” can be interpreted as signatures of phase transitions, which is further confirmed by the accompanying structural changes highlighted in the next section.

Except for a few data points, in particular around the second transition point, the two sets of points coincide within their error margin. Given the difficulty of equilibration in glass-formers, especially in the presence of critical fluctuations, we deem this result quite satisfying.

In the following graphs, we flag the estimated positions of the two peaks of Figure 1 with two vertical dotted lines. We denote $\epsilon_{1}$ and $\epsilon_{2}$ as the critical energy density of the first and the second peak, respectively.

**Figure 2:**
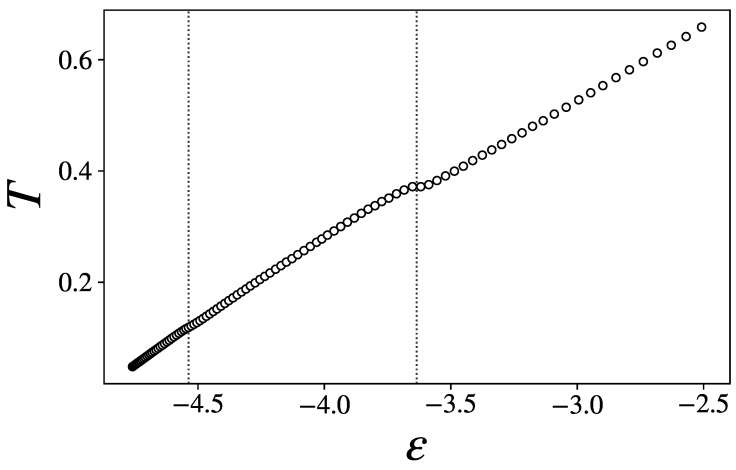
Average temperature *T* as a function of the energy density.

Figure 2 makes it clear that a plateau of the caloric curve corresponds to $\epsilon_{2}$, indicating the occurrence of latent heat in correspondence with an internal arrangement of the Lennard-Jones mixture; the higher energy transition is thus a first-order transition. On the other hand, a barely sensible inflection of the curve corresponds to $\epsilon_{1}$.

The Ehrenfest classification of order transitions relies on the loss of analyticity of Helmoltz free energy. However, the relevant thermodynamic potential in the microcanonical ensemble is the entropy, which is widely regarded as a quantity of deeper physical and mathematical meaning. Yet, after (13), the microcanonical specific heat can be rewritten as

(15) $$c_{v}\left(\epsilon \right)=-{\left(\frac{\partial S}{\partial E}\right)}^{2}{\left(\frac{\partial^{2}S}{\partial E^{2}}\right)}^{-1},$$

emphasizing that the observed singular behavior of $c_{v}$ can, in principle, originate from an analogous singularity of the first-order derivative of the entropy, or from its second-order derivative approaching zero. Furthermore, while, in the canonical ensemble, the average specific energy 〈ϵ〉(T), where ϵ=E/N is the energy density, usually displays clear critical behaviors at the transition temperature, the microcanonical ensemble inverse temperature $\beta \left(\epsilon \right)=\frac{1}{T(\epsilon )}$ is often much less sensitive.

Motivated by these observations, in [23,31,32,33,34], novel methods of classification of phase transitions in the microcanonical ensemble were proposed, relying on the analysis of inflection points of the derivatives of the entropy. In fact, in the absence of a phase transition, all derivatives of *S* of even order are strictly concave, and those of odd order strictly convex.

**Figure 3:**
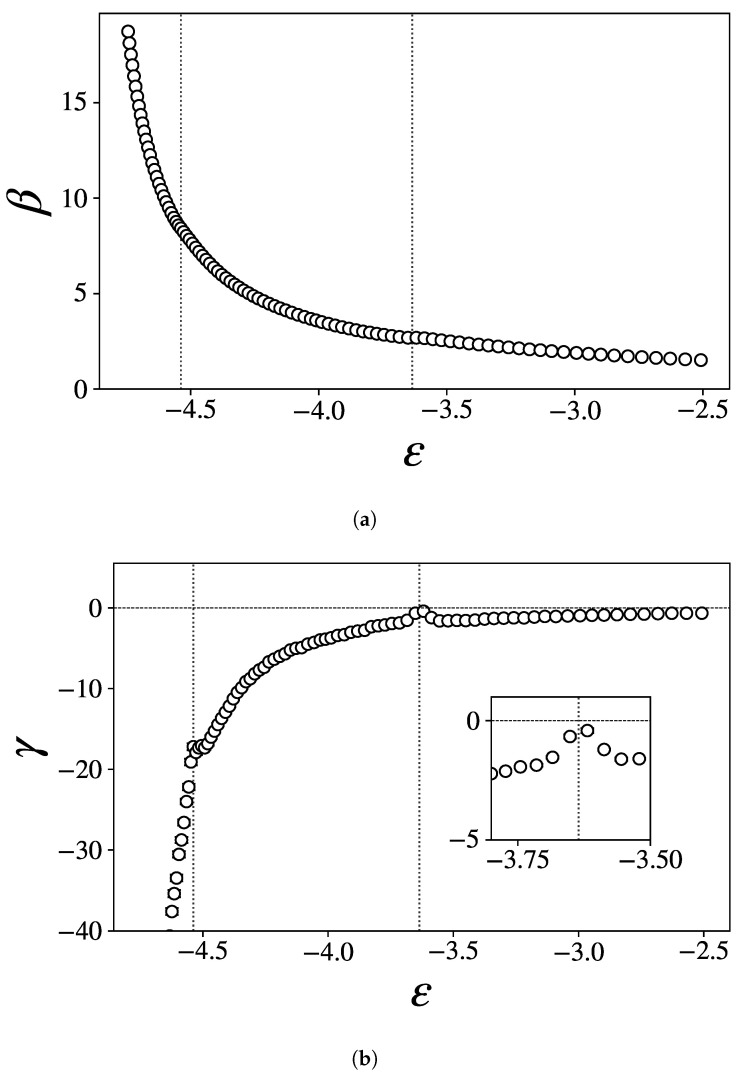
The first two derivatives of the entropy as functions of the energy density ϵ: (**a**) β, (**b**) γ. The inset in (**b**) is a zoom on the high-energy peak. A few monotonously increasing low-energy data points have been cut from (**b**) for the sake of clarity.

In Figure 3, we show the two lowest-order derivatives of *S* with respect to ϵ, namely,

(16) $$\begin{array}{cc} \beta (\epsilon ) & =\frac{\partial S}{\partial E} \end{array}$$

(17) $$\begin{array}{cc} \gamma (\epsilon ) & =\frac{\partial^{2}S}{\partial E^{2}}=\frac{\partial \beta }{\partial E} \end{array}$$

To retrieve these quantities, we departed from the kinetic temperature *T* straightforwardly obtained from simulation and differentiated β=1/T with respect to the energy.

β remains convex on the whole range of energies considered, but exhibits visible backbendings at $\epsilon_{1}$ and $\epsilon_{2}$. γ(ϵ) shows local maxima at both transition points. Around $\epsilon_{2}$, it is very close to zero, even reaching the positive region if the error is taken into account. This suggests again the occurrence of a first-order phase transition at this critical energy, according to the classification of [33]. The critical behavior of $c_{v}=-\beta^{2}/\gamma$ thus evidently originates from γ approaching zero.

These results are remarkably similar to those displayed in [32,34] for the solid–solid and solid–liquid transition of an elastic flexible polymer.

Note that, even within the thermodynamic limit, the specific heat is not expected to diverge at the glass transition critical temperature. The critical exponent α has indeed been found to be negative in various studies, e.g., in spin glass systems [35,36,37]. A more rigorous analysis of this transition, accompanied by the computation of the critical exponents, would require the study of a whole range of different system sizes, allowing for a finite-size scaling analysis [38]. One should, however, be very careful when performing said analysis as, for instance, the microcanonical specific heat has been shown to exhibit non-monotonous behaviors as a function of the system size [39]. Such analysis is outside the scope of the present work, in which we deem it sufficient to have located the transition points thanks to the anomalous behaviors of $c_{v}$ that can already be observed at very small *N*. In the following section, the occurrence of three distinct phases is confirmed by a further analysis of the configurational properties of the system.

#### 3.2.2. Translational and Orientational Order

**Figure 4:**
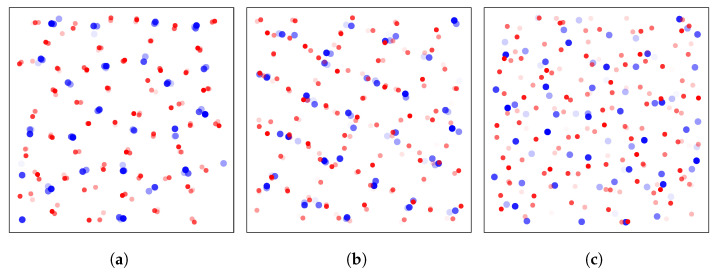
Instantaneous sample configurations projected onto a arbitrary planes, at energy density (**a**) ϵ≈−4.70, (**b**) ϵ≈−4.39, and (**c**) ϵ≈−2.51. Particles of species 1(2) are represented in large blue and small red circles, respectively.

Local ordering emerges at low energies, as exemplified by the projective views of Figure 4, where structures appear at low ($\epsilon <\epsilon_{1}$) and intermediate ($\epsilon_{1}<\epsilon <\epsilon_{2}$) energies. Depending on the projection axis, aperiodic pentagonal arrangement or square-shaped seemingly periodic structures are visible in both these regimes. However, such rough observations are very unreliable, because the visibility of ordered structures is highly dependent on the chosen projection axis. Moreover, despite this apparent order, our samples are far from crystallization and still exhibit a high degree of disorder that makes it very inconvenient for the human eye to distinguish particular geometries.

**Figure 5:**
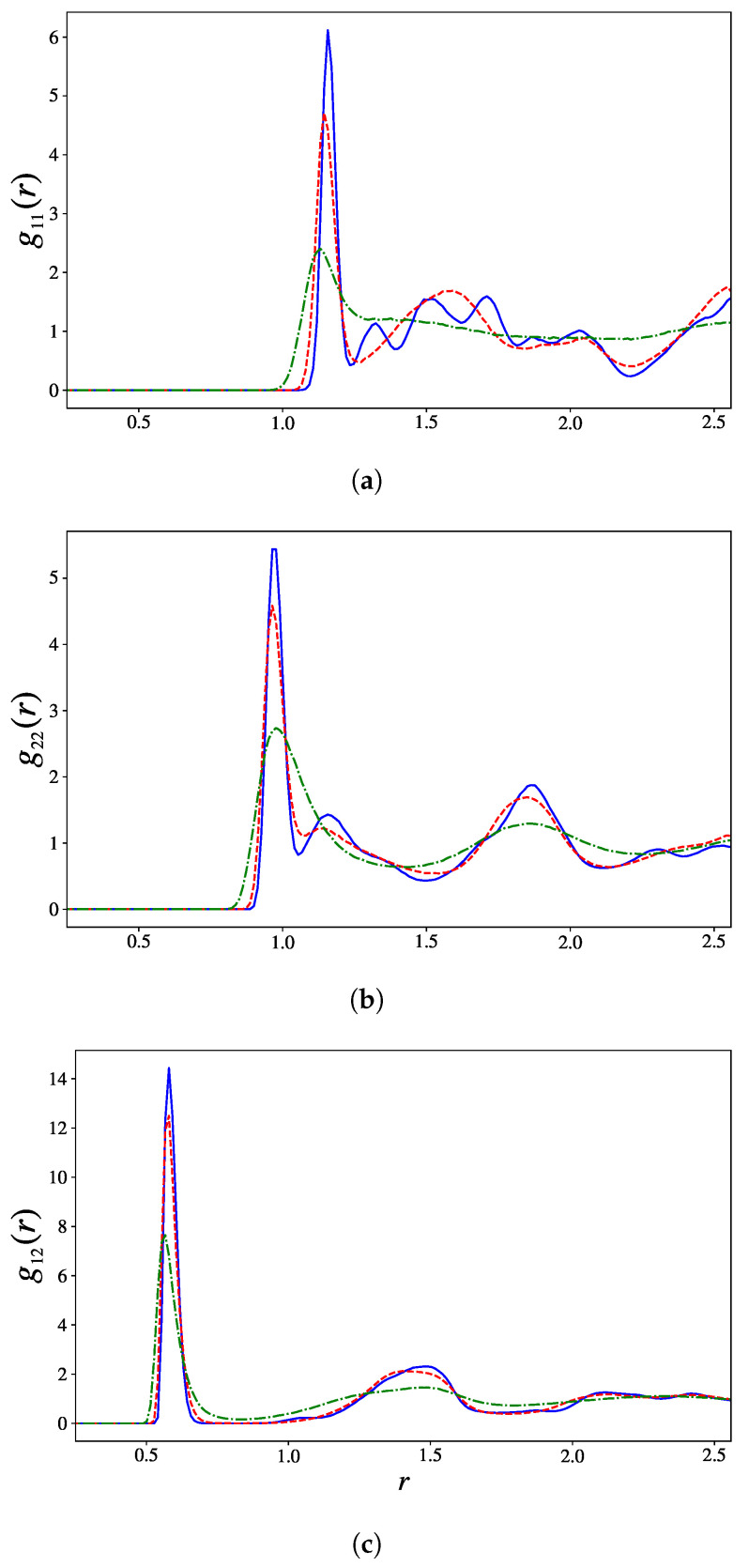
Radial pair distribution function for (**a**) 1–1 bonds, (**b**) 2–2 bonds, and (**c**) 1–2 bonds. The continuous blue lines, dashed red lines and dotted-dashed green lines correspond to systems with ϵ=−4.7016, ϵ=−4.39388 and ϵ=−2.50785, respectively.

The observation of a local order is confirmed by the profiles of the partial radial pair distribution functions $g_{\alpha \beta }\left(r\right)$ displayed in Figure 5. At high energy, these functions are in good agreement with the results obtained in [29] for T=0.39, corresponding to an energy just above $\epsilon_{2}$. At low energy, the first peak of the three functions becomes sharper, and a few other *bumps* appear at larger distances in $g_{11}\left(r\right)$ and $g_{22}\left(r\right)$. We observe the appearance of several new peaks of the density profile. This is a clear signature of the emergence of translational order. Ill-formed periodic profiles emerge at low energy, in particular in $g_{11}\left(r\right)$, indicating partial crystallization.

To further characterize the nature of these configurational changes, we inspect the bond-orientational order parameters $Q_{l}$, first defined in [40] to characterize crystalline order in Lennard-Jones liquids. For a given l∈N, it writes

(18) $$Q_{l}=\sqrt{\frac{4\pi }{2l+1}\sum_{m=-l}^{l}{\left|Q_{lm}\right|}^{2}},$$

with

(19) $$Q_{lm}=\frac{1}{n_{B}}\sum_{(i,j)\in B}Y_{lm}\left(\theta \left(r_{ij}\right),\varphi \left(r_{ij}\right)\right)\;,$$

where *B* is the considered set of bonds, $n_{B}$ its cardinality, $\theta (r_{ij})$ and $\varphi (r_{ij})$ are, respectively, the azymutal and polar angles of the bond vector $r_{ij}$ in a fixed reference frame, and the $Y_{lm}$ are spherical harmonics.

Two particles i,j are considered *bonded* if $r_{ij}<c_{b}$, where $c_{b}$ is an arbitrary cutoff. As is often prescribed [41,42,43], we set $c_{b}$ to be the approximate position of the second minimum of the radial distribution functions right after the first peak (displayed in Figure 5).

**Figure 6:**
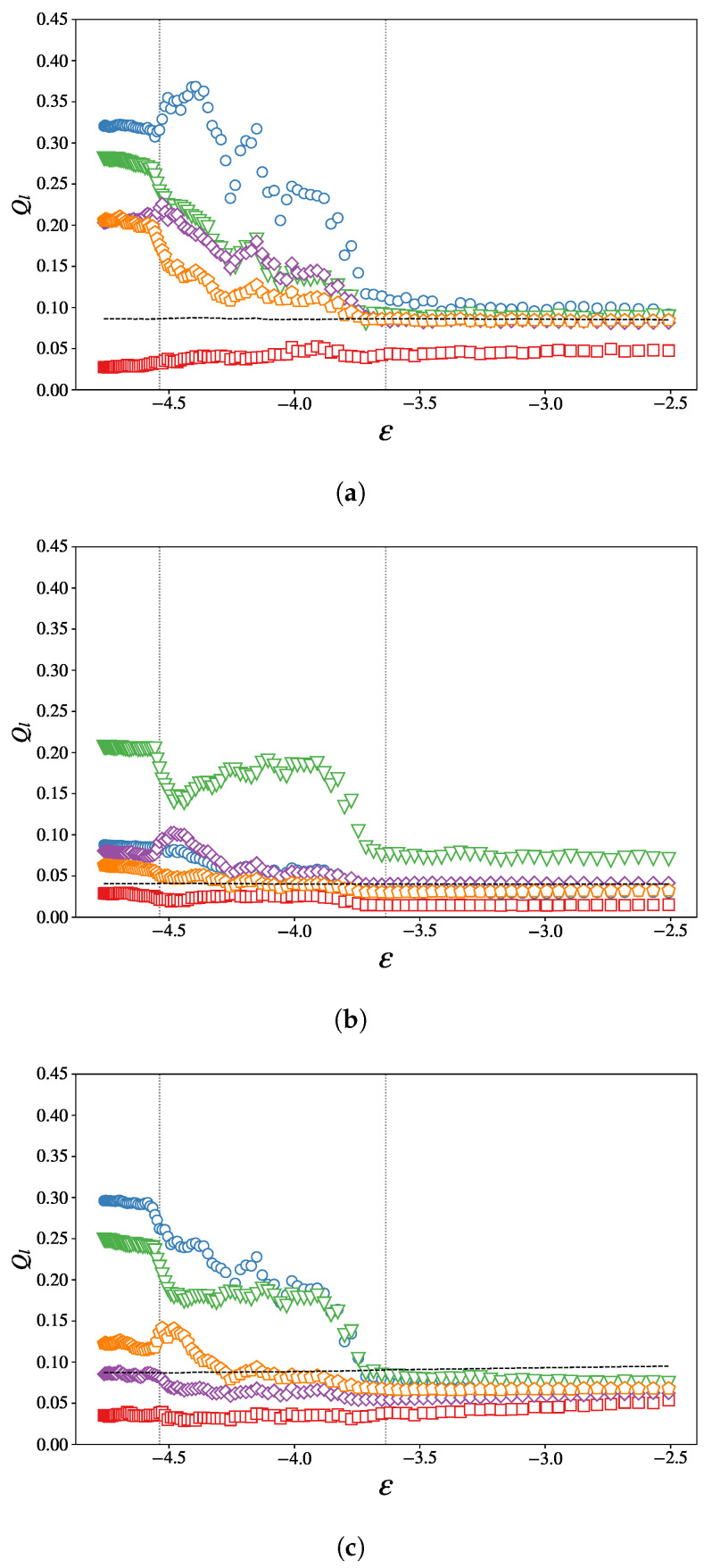
Bond-orientational order parameters $Q_{l}$ as a function of the energy density ϵ=E/N for (**a**) 1–1 bonds, (**b**) 2–2 bonds, and (**c**) 1–2 bonds. Represented are the parameters $Q_{2}$ (red squares), $Q_{4}$ (blue circles), $Q_{6}$ (green triangles), $Q_{8}$ (purple diamonds), and $Q_{10}$ (orange pentagons). The value $1/\sqrt{n_{B}}$, expected in a fully disordered system, is shown as a black line.

The authors of Ref. [29] showed that this model exhibits a short to medium-range order, namely a local tetrahedral ordering, coined as a *tetrahedral network*. The results displayed in Figure 6 seem to corroborate this observation, as the order parameters defined in Equation (18) exhibit clear steps at the transition energies $\epsilon_{1}$, $\epsilon_{2}$, for certain values of *l*.

Interestingly, at energy density $\epsilon_{1}$, most of $Q_{l}$ exhibits singular behaviors, whether it be a positive or a negative peak, suggesting a temporary rearrangement of the particles upon cooling.

The profiles of the functions $Q_{l}\left(\epsilon \right)$ clearly indicate the emergence, at low energy, of some kind of orientational order—that is, the repetition of some preferred angles between neighboring atoms throughout the whole sample. However, we found no correspondence between the combination of values we found for $Q_{l}$, instead finding that of a well-defined known crystalline structure, as described, e.g., in [40].

We thus conclude that our samples underwent frustrated crystallization, with different types of arrangement competing in the route to minimize the potential—most likely, considering the mapping given in [40], a mixing between fcc, hcp and icosahedral orders. This kind of behavior has been proposed to be a mechanism at the origin of the glass transition [15]. This competition could indeed explain the dramatic dynamical slowing down associated to this class of transitions, which we observed, in this study, in the form of a tremendously long equilibration time.

### 3.3. Topological Changes

Finally, in correspondence with these two transitions, we also found important changes in the geometrical properties of the PLS.

**Figure 7:**
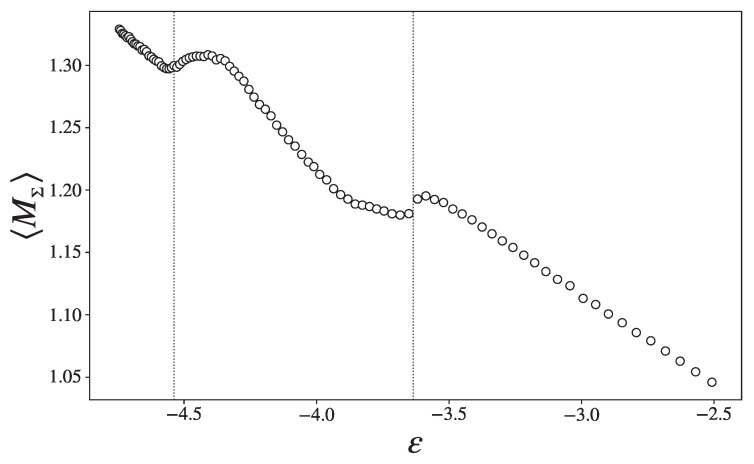
Total mean curvature as a function of the energy density.

Figure 7 shows, in correspondence with the two $c_{v}$-peaks, inflection points of the total mean curvature of the submanifold $\Sigma_{\phi }$, indicating a change in the landscape of this hypersurface. These inflection points are clear discontinuities, a sharp corner in $\epsilon_{1}$, and a step in $\epsilon_{2}$.

**Figure 8:**
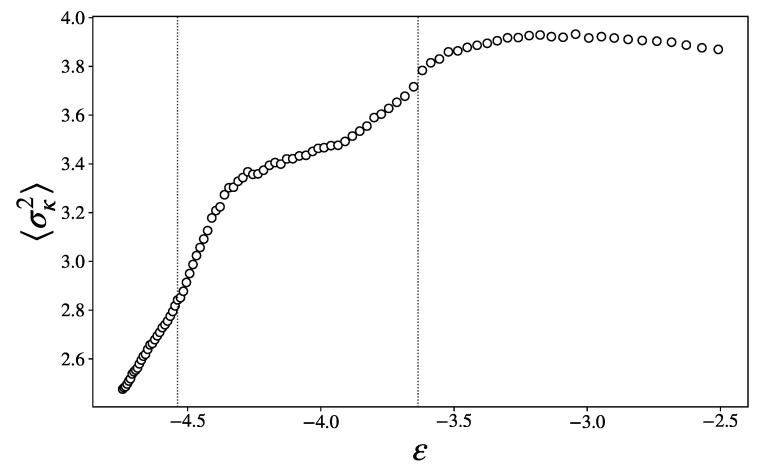
Dispersion of the principal curvatures as a function of the energy density.

The average variance of the principal curvatures, shown in Figure 8, exhibit clear changes in slope at these transition points, providing a strong indication of a change in the topology of $\Sigma_{\phi }$, according to Pinkall’s theorem (5). Specifically, such a change is necessarily due to a change in the values of the Betti numbers, and hence in the topological properties of $\Sigma_{\phi }$; though the precise nature of these changes is not accessible to our analysis, we can expect that, from the high-energy chaotic phase to the low-energy glass phase, $\Sigma_{\phi }$ loses connectivity and the system is more easily confined to restricted regions of state space.

**Figure 9:**
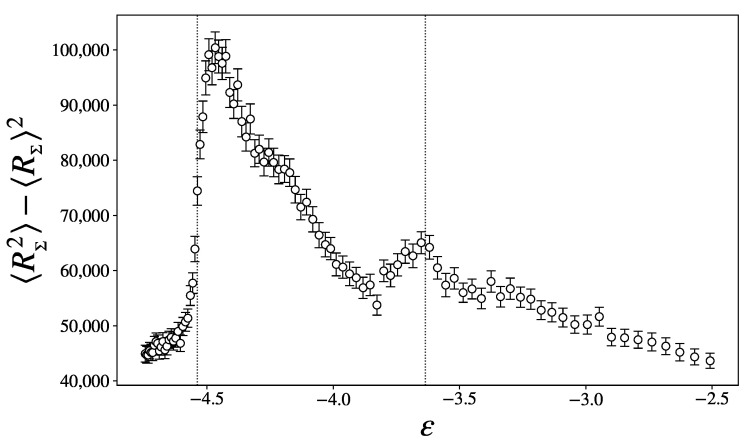
Variance of the scalar curvature as a function of the energy density.

Finally, the variance of the scalar curvature, shown in Figure 9, jumps in $\epsilon_{1}$, exhibiting a wide peak in the intermediary region, and a second, smaller peak in $\epsilon_{2}$. As per Overholt’s theorem (6), this quantity is an upper bound to the alternate sum of the Betti numbers. These sharp changes are thus indications of major topological changes in the PLS.

All of these observations suggest that there are indeed important changes in the topology of $\Sigma_{\phi }$ at play in correspondence with these two critical points of $c_{v}$. In this intermediary region, the overall shape of the manifold $\Sigma_{\phi }$ most probably undergoes dramatic changes. These changes correspond to the rearrangement of particle configurations in pseudo-crystalline orders upon cooling.

## 4. Discussion

In this work, we addressed the glass transition phenomenon using a novel approach, considering its relation to topological changes in the PLS. Using a Monte Carlo algorithm, we studied the equilibrium properties of a glass-forming system.

Although the glass transition is often considered a fundamentally dynamical phenomenon, we found several indications that, despite reaching equilibrium, the system did not fully crystallize but still exhibited clear signatures of phase transitions. This is due to the frustrated nature of the model, a Lennard-Jones binary mixture with competing interactions, which lacks a well-defined crystalline phase at the studied density.

To overcome ergodicity breaking, we employed advanced numerical techniques that enabled the system to “jump” between confined energy minima. This, combined with the absence of a crystalline phase in this frustrated system, allowed us to conduct equilibrium measurements of the glass phase using importance sampling of the state space.

Through microcanonical analysis of the entropy derivatives, we identified two distinct transitions: a second-order transition at low energy and a first-order transition at higher energy. We propose that the first-order transition corresponds to a glass-liquid transition, while the second-order one represents a reconfiguration of the glass into different local orderings. This is supported by our finding of significant changes in the radial pair distribution function and bond-orientational order parameters at the transition points. These results deviate from what would be expected in a crystal and instead align with recent studies [14,15], suggesting that the glass transition can be understood in terms of frustration on the path to crystallization.

Finally, in agreement with the topological theory of phase transitions, we observed that several quantities closely related to the topology of the PLS exhibit inflection points and discontinuities at the transition energies. This study introduces a new perspective on the phenomenon of vitrification.

Future research directions include repeating our experiment with different system sizes to perform finite-size scaling, confirm our findings, and calculate critical exponents. Additionally, identifying crystalline seeds and performing localized measurements of the studied quantities would allow for the computation of spatial correlation functions. These correlations are expected to diverge at large *N* (see [15]). Moreover, this approach would clarify how sensitive the topological analysis is to scalin—in other words, whether the well-known degeneracy of energy minima in the glass phase is a global phenomenon or if the system locally stabilizes into well-defined energy minima.

## Data Availability

The raw data supporting the conclusions of this article will be made available by the authors on request.

## Appendix A. Monte Carlo Methods

Simulating glass formers is a notoriously complex task due to the large number of energy minima in the glass phase, where systems tend to get trapped. To sample the energy surface effectively, we use a Monte Carlo algorithm (MC), a common choice for equilibrium simulations. It is a class of ergodic algorithms, relying on random displacements rather than exact dynamics, allowing them to avoid getting stuck in potential wells. Unlike molecular dynamics, MC methods do not require solving equations of motion, offering significant computational speedup.

In a sound MC algorithm, provided the selection probability of a given configuration is uniform, the maximal acceptance rate satisfying detailed balance writes [44]

(A1) $$A\left(\Gamma_{\mu }\to \Gamma_{\nu }\right)=\min\left\{1,\frac{p(\Gamma_{\mu })}{p(\Gamma_{\nu })}\right\}\;,$$

where $\Gamma_{\mu }$ and $\Gamma_{\nu }$ denote two distinct microstates, and *p* their probability weights in the chosen Gibbs ensemble statistics.

Microcanonical MC are uncommon in the literature, and less straightforward in their implementation because, in this ensemble, the system is strictly constrained to a surface of constant energy and thus evolves in a subspace of null measure in Σ.

To address this difficulty, we use the separability of the partition function. Since, at equilibrium, momenta trivially follow the Boltzmann distribution, random momentum changes at each step of the algorithm are unnecessary. Ignoring these degrees of freedom leaves the kinetic energy available as a free parameter. We use it instead as a “demon” to compensate the changes in potential energy and maintain the total energy of the system at the desired value.

The microcanonical probability density for a given microstate $\Gamma_{\mu }$ at energy *E* is given by [24,45,46]

(A2) $$p_{E}\left(\Gamma_{\mu }\right)\propto \Theta \left(E-\phi_{\mu }\right){\left[E-\phi_{\mu }\right]}^{\left(3N/2-1\right)}\;,$$

where $\phi_{\mu }$ is the potential energy of the configuration $\Gamma_{\mu }$ and Θ(x) is the Heaviside function.

Let us now consider a transition $\Gamma_{\mu }\to \Gamma_{\nu }$. For any physically meaningful initial state, we must have $E\geq \phi_{\mu }$, hence $\Theta \left(E-\phi_{\mu }\right)=1$. Equation (A1) becomes

(A3) $$A\left(\Gamma_{\mu }\to \Gamma_{\nu }\right)=\min\left\{1,\Theta \left(E-\phi_{\nu }\right){\left[\frac{E-\phi_{\nu }}{E-\phi_{\mu }}\right]}^{\left(3N/2-1\right)}\right\}$$

To fully take advantage of the MC framework, we implemented moves that allow then system to escape potential wells.

First, we introduced particle swapping, first proposed in [30]. At each MC step, with a probability that we (arbitrarily) set to p=0.1, one particle of each species is chosen at random, both their positions are swapped, and then displaced according to the usual random displacement rule; with probability (1−p), a conventional MC step is performed. Afterwards, the move is accepted according to the usual acceptance rate (A3).

We also resorted to a *replica exchange* (RE) algorithm, often called *parallel tempering* in the literature. We thereafter use the term RE, renouncing the conventional terminology, which is somewhat misleading here, given that the control parameter of our simulations is energy rather than temperature.

*M* replicas of the system are simulated in parallel, each at a different energy. Every 2000 sweeps (a sweep equates N MC steps), an attempt is made to exchanges replicas of neighboring energies.

The acceptance rate for the exchange of a replica *i* of energy $E_{i}$ in state $\Gamma_{\mu }$ with a replica i+1 of energy $E_{i+1}$ in state $\Gamma_{\nu }$ is straightforwardly deduced from detailed balance

(A4) $$\begin{array}{cc} A_{i,i+1}\left(\Gamma_{\mu },\Gamma_{\nu }\to \Gamma_{\nu },\Gamma_{\mu }\right) & =\min\left\{1,\frac{p_{i}\left(\Gamma_{\mu }\right)p_{i+1}\left(\Gamma_{\nu }\right)}{p_{i}\left(\Gamma_{\nu }\right)p_{i+1}\left(\Gamma_{\mu }\right)}\right\}\\ \; & =\min\left\{1,\Theta \left(E_{i}-\phi_{\nu }\right){\left[\frac{\left(E_{i}-\phi_{\nu }\right)\left(E_{i+1}-\phi_{\mu }\right)}{\left(E_{i}-\phi_{\mu }\right)\left(E_{i+1}-\phi_{\nu }\right)}\right]}^{\left(3N/2-1\right)}\right\} \end{array}$$

As $A_{i,i+1}∼o\left(e^{N}\right)$, its large *N* limit is relevant to evaluate the performance of our RE scheme, even in our relatively small system. We easily derive

(A5) $$\begin{array}{c} \lim_{N\to \infty }A_{i,i+1}\left(\Gamma_{\mu },\Gamma_{\nu }\to \Gamma_{\nu },\Gamma_{\mu }\right)=\Theta \left(\phi_{\mu }-\phi_{\nu }\right), \end{array}$$

which, in practice, were valid throughout all of our simulations.

Clearly, the overall efficiency of the RE algorithm strongly depends on the overlaps of the potential energy distributions $p_{E}\left(\phi \right)$, and therefore on the number of replicas *M*, and on the choice of energy array ${\left\{E_{i}\right\}}_{i=0,\cdots ,M-1}$. We found that it is not enough, for a fixed *M*, to choose linear or exponential energy spacings. Some energies’ regions indeed behave as bottlenecks, through which replicas cannot diffuse efficiently enough, thus requiring finer optimization of the energy array.

We thus resorted to a simple algorithm, described in [47,48], in the context of canonical ensemble simulations, and coined in this context the *energy method* (which, would be the *potential method*); the adaptation to the microcanonical case is straightforward. Given two fixed extremal energies $E_{0},\;E_{M}$ and the number *M* of replicas we wish to simulate, it consists of finding the M−2 intermediate energies such that the exchange rate between adjacent replicas is uniform. In other words, we observe convergence towards the fixed points $\langle A_{i,i+1}\rangle =\langle A_{i,i-1}\rangle ,\;\forall i=0,\cdots ,M$. In principle, the closer we are to these fixed points, the more efficient the RE algorithm is.

In [47,48] (thus, in the canonical context), the average acceptance rates were computed by simply inserting the average energies 〈E〉(T) into the Boltzmann weight corresponding to the transition. In the microcanonical context, we cannot proceed as straightforwardly, since the acceptance rate roughly reads as (A5); simply inserting the average potential energy 〈Φ〉(E) would result in null or much too small average acceptance rates.

To get around this issue, we simply take into account the overlap of potential energy distributions instead of the mere average; that is,

(A6) $$\langle A_{i,i+1}\rangle =\int_{-\infty }^{E_{i+1}}d\phi^{\prime }\;\int_{-\infty }^{E_{i}}d\phi \;p_{E_{i}}\left(\phi \right)p_{E_{i+1}}\left(\phi^{\prime }\right)A_{i,i+1}\left(\phi ,\phi^{\prime }\to \phi^{\prime },\phi \right).$$

Then, applying the approximation (A5) greatly accelerates the algorithm, as it avoids much exponentiation and reduces the interval in which the above integral is computed. We end up with

(A7) $$\begin{array}{cc} \langle A_{i,i+1}\rangle & \approx \int_{-\infty }^{E_{i+1}}d\phi^{\prime }\;\int_{-\infty }^{E_{i}}d\phi \;p_{E_{i}}\left(\phi \right)p_{E_{i+1}}\left(\phi^{\prime }\right)\Theta \left(\phi -\phi^{\prime }\right)\\ \; & =\int_{-\infty }^{E_{i+1}}d\phi^{\prime }\;\int_{\phi^{\prime }}^{E_{i}}d\phi \;p_{E_{i}}\left(\phi \right)p_{E_{i+1}}\left(\phi^{\prime }\right). \end{array}$$

To estimate the distributions $p_{E}\left(\phi \right)$, we initially set the energy array following a power law, i.e., $E_{i}=E_{min}{\left(\frac{E_{max}}{E_{min}}\right)}^{\frac{i}{M-1}}$ with i=0,⋯,M−1, and run MC simulations until we obtain a reasonable energy-temperature dependence. We then computed and interpolated the average $\mu_{\phi }\left(E\right)$ and standard deviation $\sigma_{\phi }\left(E\right)$ of ϕ as functions of *E*, which enabled us to roughly estimate the distributions $p_{E}\left(\phi \right)$ as plain Gaussian distributions.

The integrals (A7) were finally computed numerically using a discrete set of $10^{3}$ potential energies in the range $\mu_{\phi }\left(E_{i}\right)-2\sigma_{\phi }\left(E_{i}\right)\leq \phi \leq \;\mu_{\phi }\left(E_{i+1}\right)+2\sigma_{\phi }\left(E_{i+1}\right)$.

**Algorithm A1:** *E*-range optimization through *U*-overlap method

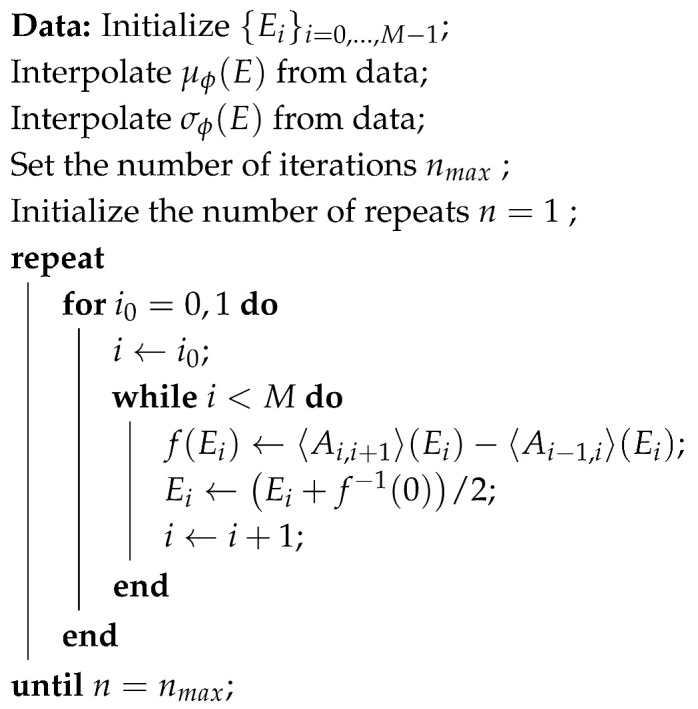

## Appendix B. Initial Configuration, Periodic Boundary Conditions and Cutoff

The system was simulated in a cubic box of volume $L^{3}$.

The continuous potential (12) depends on negative powers of the interatomic distances $r_{ij}$ and is repulsive at short range. When some $r_{ij}$ become too small, the energy thus diverges. Since our simulations were performed at high density (ρ>1), totally random initial configurations typically result in very high energies, which cannot be corrected by the choice of *K*, since the latter is a positive quantity.

To get around this issue and have sufficient control over the initial ϕ, we had to implement initial conditions with a high degree of symmetry. Specifically, we initially arranged the particles in a cubic lattice configuration, where each particle’s first neighbors were of the other species. Then, we randomly replaced particles of species 1 with particles of species 2 until we reached the desired species ratio.

To avoid undesired boundary effects, we implemented, as is customary, periodic boundary conditions (PBCs).

Note that the PBCs entail a periodic pattern in the particles’ spatial distribution. Smooth cutoffs must therefore be implemented, not only for performance purposes, but also to avoid this unwanted symmetry introduced by the PBCs. Since we were aiming to compute quantities derived from both the gradient and the Hessian of ϕ(Γ), we accordingly needed cutoffs that were continuous up to the second order.

The non-monotonic, interspecies potential of Equation (12) is redefined as

(A8) $$\left\{\begin{array}{cc} \phi_{12}\left(r\right)=4\epsilon_{12}\left(\sigma_{12}^{12}r^{-12}-\sigma_{12}^{6}r^{-6}\right) & ,\;\;0<r\leq r_{c}^{\left(12\right)}\\ \phi_{12}\left(r\right)=C{\left(r-r_{m}^{\left(12\right)}\right)}^{4}+D{\left(r-r_{m}^{\left(12\right)}\right)}^{3} & ,\;\;r_{c}^{\left(12\right)}<r\leq r_{m}^{\left(12\right)}\\ \phi_{12}\left(r\right)=0 & ,\;\;r_{m}^{\left(12\right)}<r, \end{array}\right.$$

On the other hand, the monotonic, intra-species potentials are redefined as

(A9) $$\left\{\begin{array}{cc} \phi_{\alpha \alpha }\left(r\right)=4\epsilon_{\alpha \alpha }\sigma_{\alpha \alpha }^{12}r^{-12}+A_{\alpha } & ,\;\;0<r\leq r_{c}^{\left(\alpha \alpha \right)}\\ \phi_{\alpha \alpha }\left(r\right)=B_{\alpha }{\left(r-r_{m}^{\left(\alpha \alpha \right)}\right)}^{3} & ,\;\;r_{c}^{\left(\alpha \alpha \right)}<r\leq r_{m}\\ \phi_{\alpha \alpha }\left(r\right)=0 & ,\;\;r_{m}^{\left(\alpha \alpha \right)}<r, \end{array}\right.$$

where α=1,2.

Following [49], we set $r_{c}^{\left(12\right)}={\left(\frac{7}{26}\right)}^{1/6}\sigma_{12}$, i.e., the distance such that $\phi_{12}^{\prime \prime }\left(r_{c}^{\left(12\right)}\right)=0$. Though this condition becomes meaningless for the monotonic potentials, we also, arbitrarily, chose $r_{c}^{\left(\alpha \alpha \right)}={\left(\frac{7}{26}\right)}^{1/6}\sigma_{\alpha \alpha }$.

From there, requiring the continuity—up to the second derivative—of these potential functions at the first cutoff distance $r_{c}$ straightforwardly implies the values of the other constants of Equations (A8) and (A9).
